# A Case of Strabismus Fixus With Extremely Long Axial Length Results in Improvement of Limitation of Abduction by the Yokoyama Procedure

**DOI:** 10.7759/cureus.58194

**Published:** 2024-04-13

**Authors:** Chihiro Koiwa, Takashi Negishi, Fumika Sakemi, Atsuhide Takesue, Toshiyuki Yokoyama

**Affiliations:** 1 Department of Ophthalmology, Juntendo University Faculty of Medicine, Tokyo, JPN; 2 Department of Ophthalmology, Juntendo University Nerima Hospital, Tokyo, JPN

**Keywords:** medial rectus recession, yokoyama procedure, myopic strabismus fixus, acquired strabismus, myopia

## Abstract

Acquired strabismus in high myopia is typically fixed in the positions of adduction and depression, with restrictions in both abduction and elevation. As a treatment for myopic strabismus fixus, the Yokoyama procedure is effective. We report a case of strabismus fixus with a long axial length (34 mm), in which abduction limitation was improved by the Yokoyama procedure with medial rectus recession. A 68-year-old woman was referred for strabismus fixus in her right eye. Her right eye was fixed in the positions of adduction and depression, with restrictions in both abduction and elevation. The axial length of her right eye was extremely long 33.97mm. Magnetic resonance imaging (MRI) showed that the posterior eyeball of her right eye had dislocated out of the superotemporal muscle cone, and she was diagnosed with strabismus fixus with high myopia. She underwent the Yokoyama procedure in her right eye, and medial rectus recession was performed at the same time because abduction limitations remained at the end of the Yokoyama procedure. After surgery, there was a small residual esotropia, but abduction beyond the midline was possible, and the patient's satisfaction was high. A combination of the Yokoyama procedure and medial rectus recession for a patient with myopic strabismus fixus with long axial length resulted in good improvement of ocular misalignment and limitation of abduction.

## Introduction

The myopic population is increasing worldwide [1]. Myopia is associated with ocular disorders such as cataracts, myopic maculopathy, retinal detachment, retinoschisis, glaucoma, and myopic strabismus [1-3]. Acquired strabismus in high myopia is typically fixed in the positions of adduction and depression, with restrictions in both abduction and elevation [3]. As a treatment for myopic strabismus fixus, the Yokoyama procedure, which unites the superior and lateral rectus muscles approximately 15 mm posterior to their insertions, is effective [4]. We report a case of strabismus fixus with an extremely long axial length (34 mm), in which abduction limitation was improved by the Yokoyama procedure with medial rectus recession.

## Case presentation

**Figure 1:**
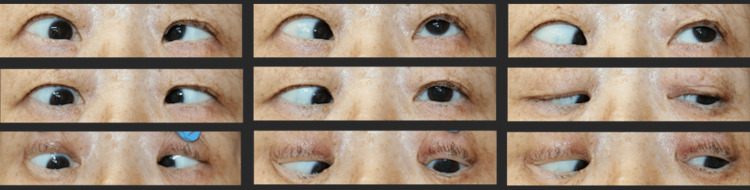
Nine gaze photographs before the surgery Her right eye was fixed in the positions of adduction and depression, with restrictions in both abduction and elevation.

A 68-year-old woman was referred for strabismus fixus in her right eye. Her right eye was fixed in the positions of adduction and depression, with restrictions in both abduction and elevation (Figure 1).

**Figure 2:**
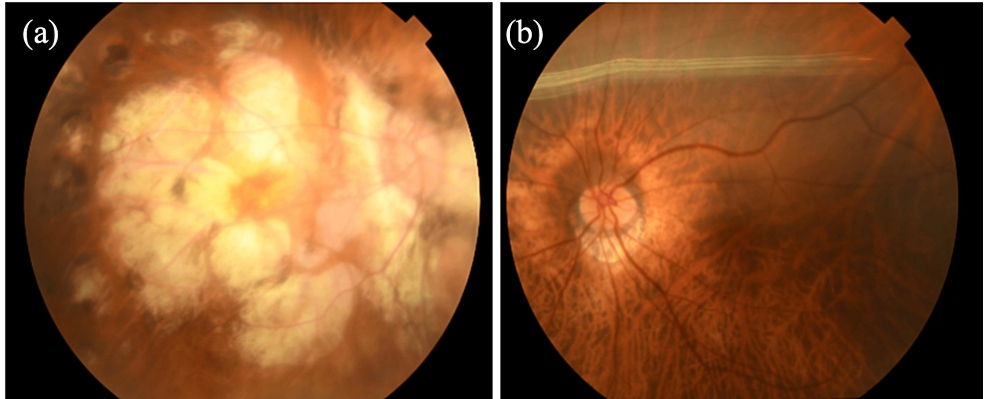
Fundus photographs The right eye is (a) and the left is (b). Extensive chorioretinal atrophy was observed in her right eye.

She had approximately 113Δ (40+50Δ in front of each eye) esotropia in the right eye with left eye fixation in the primary position by the Krimsky test. Her corrected visual acuity was 0.02 in the right eye and 0.5 in the left eye, and the objective refraction of her right eye was unmeasurable because of the position of her right eye. Both of her eyes had cataracts and corneal scars after a radial keratotomy. Extensive chorioretinal atrophy is observed in her right eye (Figure 2 (a)).

**Figure 3:**
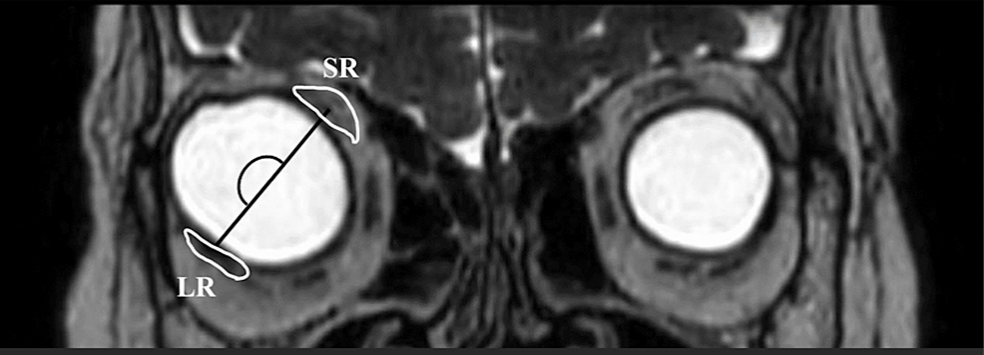
MRI image before the surgery An MRI showed that the posterior eyeball of her right eye had dislocated out of the supratemporal muscle cone. The dislocation angle between the superior rectus (SR) and lateral rectus (LR) was 181° in the right eye.

The axial length of her right eye was extremely long 33.97mm. The axial length of her left eye was 27.64 mm. Magnetic resonance imaging (MRI) showed that the posterior eyeball of her right eye had dislocated out of the superotemporal muscle cone, and she was diagnosed with strabismus fixus with high myopia (Figure 3).

**Figure 4:**
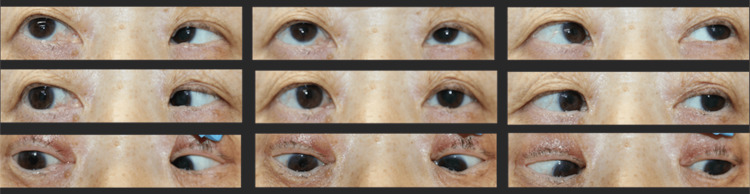
Nine gaze photographs one year after the surgery Small residual esotropia still existed but resulted in good improvement of ocular misalignment and limitation of abduction.

**Figure 5:**
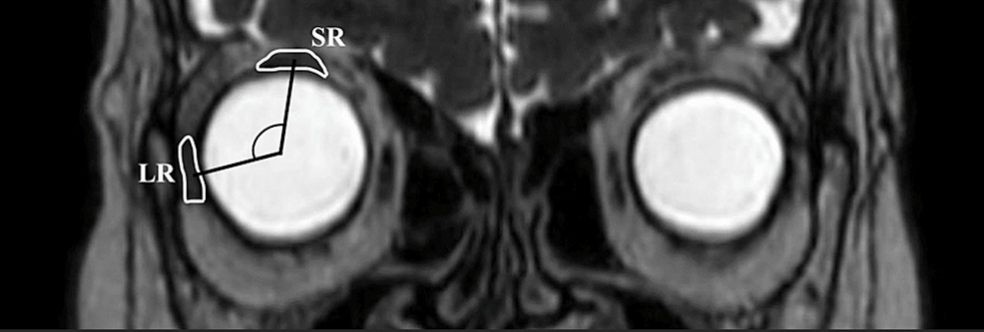
MRI image after the surgery After surgery, the dislocation angle improved to 104°.

The Yokoyama procedure was first performed on her right eye under general anesthesia. A 5-0 polyester thread was applied to the muscle belly 15 mm posterior to the insertion of the superior rectus muscle, then the lateral rectus muscle was threaded 15 mm posterior to the insertion as well, and both muscles were pulled together and sutured. A 5 mm medial rectus recession was performed simultaneously because abduction limitations remained at the end of the Yokoyama procedure. After surgery, there was a small residual esotropia (approximately 20Δ esotropia by Krimsky test), but abduction beyond the midline was possible, and the patient's satisfaction was high (Figures 4, 5).

## Discussion

As treatment for myopic strabismus fixus, the Yokoyama procedure was effective in restoring the dislocated globe into the muscle cone and improving both ocular motility and deviation [5]. The mean axial length of myopic strabismus fixus was reported to be 28.9 ±2.03 mm by Nakao et al., 30.5 ± 3mm by Maiolo et al., and 32 ± 5mm by Demer et al. [6-8]. The axial length, in this case, was 33.97 mm, which was relatively long even among severe myopic strabismus fixus.

Yamaguchi et al. reported mean angles of dislocation of the globe were 179.9 ± 30.8° in myopic strabismus fixus, suggesting that half of the cross-section of the globe was located outside the muscle cone [5]. In addition, it was reported at 132 ± 14° by Nakao et al., 167.5 ± 12.9° by Maiolo et al., and 121 ± 7° by Demer et al. [6-8]. The mean angle of dislocation of the globe without strabismus fixus was 102.9 ± 6.8° [5]. In our case, the dislocation angle was 181°, which indicated that surgery was highly effective in this case.

Wabbels et al. reported that an additional medial rectus recession had no additional effect on postoperative outcomes compared with the pure Yokoyama procedure and may lead to overcorrections [9]. In contrast, Yamaguchi et al. recommended recession of the medial rectus in cases that have had restricted abduction for many years because contracture of the medial rectus muscle is likely to have occurred [5]. In this case, an additional medial rectus recession was performed simultaneously because abduction limitation remained at the end of the Yokoyama procedure and resulted in good improvement of abduction despite the high long eye axis of 34 mm.

When high myopia is defined as -5.00 D or less, it is estimated that the prevalence of myopia and high myopia will increase significantly worldwide, affecting nearly 5 billion and 1 billion people, respectively, by 2050 [10]. Vitale et al. reported that the prevalence of high myopia (≤ -7.90 D) increased from 0.2% to 1.6% over 30 years [11]. Morgan et al. report that the higher prevalence of myopia, particularly in East Asian cities, is thought to be related to increased educational pressure coupled with lifestyle changes that have resulted in children spending less time outside [12]. In a group of Japanese participants (22,379), mainly in their 60s and 70s, 6.96% had strong myopia with an axial length of more than 26.0 mm in both eyes [13]. Although the axial length in the present case was particularly long, long axial length cases are not uncommon in Japan, and such cases are expected to increase in the future.

## Conclusions

A combination of the Yokoyama procedure and medial rectus recession for a patient with myopic strabismus fixus with long axial length resulted in good improvement of ocular misalignment and limitation of abduction. For patients with residual abduction limitation at the end of the Yokoyama procedure, simultaneous medial rectus muscle recession should be considered. The prevalence of high myopia is increasing substantially on a global scale. As this population ages, ophthalmologists can expect to encounter vision-threatening complications like myopic strabismus fixus more frequently. Successful outcomes in complex cases such as this one will be imperative.
